# The regulation of neuroinflammatory response after stroke by intestinal flora microorganisms

**DOI:** 10.3389/fcimb.2025.1594834

**Published:** 2025-06-23

**Authors:** Wanying Xie, Xue Yan, Xu Yang, Haitao Sun, Weimin Zhang

**Affiliations:** ^1^ College of Integrated Traditional Chinese and Western Medicine, Changchun University of Chinese Medicine, Changchun, China; ^2^ The Department of Encephalopathy, The Third Clinical Hospital Affiliated to Changchun University of Traditional Chinese Medicine, Changchun, China

**Keywords:** dysbiosis, gut microbiota, ischemic stroke, microbiota-gut-brain axis, neuroinflammation

## Abstract

Ischemic stroke (IS) is a severe central nervous system disorder characterized by high incidence, disability, mortality, and recurrence rates, along with numerous complications. The microbiota-gut-brain axis (MGBA) represents a bidirectional communication pathway between the brain and the gut, which can influence the onset and progression of IS through neural, immunoregulatory, and gut metabolite pathways. Recent preclinical and clinical evidence supports the use of fecal microbiota transplantation (FMT), probiotics and prebiotics, dietary interventions, and antibiotics as strategies to suppress neuroinflammation in IS, protect the blood-brain barrier, modulate immune responses, and improve stroke outcomes. In this review, we summarize the manifestations of innate inflammation and adaptive immunity following the onset of IS, highlight the interactions between the MGBA and post-stroke neuroinflammation, and discuss current therapeutic measures, thus providing insights for the development of novel treatment strategies in the future.

## 1 Introduction

According to a recent report by the World Stroke Organization, stroke remains the second leading cause of death and the third leading cause of combined death and disability worldwide. Stroke-related mortality accounts for 86.0% of global stroke incidence, while the prevalence of post-stroke dementia reaches 89.0% (Feigin et al., 2022). Stroke can be classified into two types: ischemic stroke and hemorrhagic stroke (Alsbrook et al., 2023). The incidence of IS is significantly higher than that of hemorrhagic stroke (Ananth et al., 2023). IS occurs due to various factors that lead to the complete or partial blockage of cerebral blood vessels, resulting in impaired blood flow, ischemia, hypoxia, necrosis of the corresponding brain tissue, and irreversible neuronal apoptosis (Jayaraj et al., 2019). Neuroinflammation accompanies the entire course of IS and plays a crucial role in the disease process. Upon the occurrence of intravascular occlusion, the inflammatory cascade is promptly activated. In the early stages of IS, damage-associated molecular patterns (DAMPs) released by injured neural cells activate immune cells in the central nervous system, with microglial activation being particularly prominent (Petrovic-Djergovic et al., 2016). Microglia are activated through the recognition of DAMPs by pattern recognition receptors on their surface, playing dual roles in both neuroprotection and neurodamage. During the initial phases of the disease, activated inflammatory microglia release a substantial quantity of inflammatory factors, which in turn activate astrocytes, disrupt the blood-brain barrier, recruit peripheral immune cells, and exacerbate brain damage (Liang et al., 2023).The current treatment for IS primarily aims to restore local blood flow. This includes acute phase interventions such as the administration of recombinant tissue plasminogen activator (r-tPA) and endovascular thrombectomy, both of which are subject to time constraints (Jovin et al., 2015; Cui et al., 2022). It is important to note that these interventions do not repair damaged cells and tissues; rather, they primarily enhance local blood circulation.

Recently, the potential therapeutic and prognostic applications of gut microbiota in stroke, mediated through the MGBA, have garnered significant attention (Singh et al., 2018; Wang et al., 2021; Zhu et al., 2021). The MGBA represents a bidirectional communication network between gut microbiota and the host, where its dysfunction, known as dysbiosis, is associated with the occurrence, progression, and therapeutic response of IS. For instance, the gut microbial metabolite trimethylamine-N-oxide (TMAO) is linked to an increased risk of IS and adverse outcomes, demonstrating a positive correlation with cerebral infarction volume (Zhu et al., 2021). Furthermore, elevated levels of TMAO can enhance the expression of pro-inflammatory cells, thereby increasing the risk of cardiovascular diseases (Haghikia et al., 2018). In contrast, the gut microbial metabolite short-chain fatty acids (SCFAs) can regulate the maturation, development, and immune responses of innate immune cells, such as microglia, and influence organ-specific autoimmunity (Erny et al., 2015). Furthermore, SCFAs can induce connectivity changes in the contralateral cortex by crossing the blood-brain barrier, thereby promoting the recovery of limb motor function after IS (Sadler et al., 2020). Drawing on previous research, this review highlights the role of gut microbiota in neuroinflammatory damage associated with IS, outlines the current understanding of this subject, and discusses the diagnostic and prognostic implications of gut microbiota in clinical trials involving IS patients, thereby enhancing the comprehension of gut microbiota’s role in post-stroke neuroinflammation.

## 2 The mechanism of interaction between IS neuroinflammation and gut microbiota

### 2.1 Inflammatory response following cerebral ischemia

#### 2.1.1 Congenital inflammatory response

The pathogenesis of IS involves multiple mechanisms, with neuroinflammatory responses playing a significant role in both the onset and prognosis of the disease. Following an IS event, the deprivation of oxygen and glucose—essential nutrients in the blood—results in the cessation of their supply to tissues. This leads to the inactivation of ion pumps and related channels, the release of excitatory neurotransmitters, and the initiation of oxidative stress reactions. Concurrently, extensive necrosis of cells and tissues occurs in the ischemic area. The necrotic cells and tissues, along with persistent oxidative stress reactions, induce the release of a substantial amount of DAMPs (Petrovic-Djergovic et al., 2016). High-mobility group box 1 (HMGB1) is classified as a damage-associated DAMPs, and its various oxidized forms are rapidly released from necrotic cells located in the ischemic core, thereby activating immune cells and initiating inflammatory responses. Additionally, HMGB1 can undergo oxidative modifications in the circulation, which further activates peripheral immune cells (Singh et al., 2016b). The release of substantial amounts of DAMPs triggers innate immune responses, including the activation of microglia and astrocytes, leading to cascading intravascular inflammatory reactions. While innate immune cells in the brain work to clear necrotic tissue, they concurrently release a significant quantity of inflammatory factors. Furthermore, the increased permeability of the blood-brain barrier, combined with the activity of peripheral immune cells, results in excessive immune activation at the lesion site, thereby exacerbating brain injury.

#### 2.1.2 Peripheral immune response

Neuroinflammation is initiated not only by innate immune cells but also by immune cells that infiltrate from the peripheral immune system. Following IS, neutrophils and monocytes are the first responders, entering the brain within 12 hours, followed by T cells and B cells, which infiltrate the brain hours to days after IS (DeLong et al., 2022). Within hours of the onset of IS, the peripheral neutrophil count increases exponentially, whereas the lymphocyte count exhibits a contrasting trend (Gill et al., 2018). In the field of acute ischemic stroke (AIS), the neutrophil-to-lymphocyte ratio has been recognized as a significant biomarker for patient prognosis. It predicts early neurological improvement following thrombolysis in patients with AIS and is associated with early neurological deterioration (Gong et al., 2021; Sharma et al., 2021). Following a stroke, monocytes migrate from peripheral tissues to the site of injury, guided by a gradient of secreted cytokines and chemokines, such as CCL2/CCR2, CX3CL1/CX3CR1, and MMP-2/9. At the injury site, these monocytes differentiate into macrophages, contributing to the inflammatory response and tissue repair (Kim and Cho, 2016). Furthermore, both the monocyte-to-high-density lipoprotein cholesterol ratio and the monocyte-to-lymphocyte ratio serve as predictive markers for IS risk. The combined application of these ratios provides more accurate diagnostic results (Liu et al., 2020a). T cells are recruited to the brain by inflammatory mediators, particularly the CD4+ Th1 and Th17 subtypes, which exacerbate the severity and outcomes of stroke. In contrast, regulatory T cells (Tregs) are beneficial for stroke recovery and are associated with improved outcomes following severe strokes (Endres et al., 2022). B cells play a critical role in modulating the inflammatory response during the acute phase of IS and are involved in the recruitment of immune cells (Wu et al., 2024). As the disease progresses into the subacute and chronic phases of IS, the function of B cells shifts from regulating inflammation to participating in tissue repair and the long-term modulation of the immune response (Malone et al., 2023). Following a stroke, both the innate and adaptive immune systems contribute to the cascade of brain injury, engaging in complex and interdependent roles that enhance the inflammatory response, clear necrotic tissue, and protect brain cells.

### 2.2 The interaction between gut microbiota and neuroinflammation

#### 2.2.1 The impact of gut microbiota diversity on neuroinflammation

The microbiota represents a complex and diverse ecosystem within the human body. The primary sites of microbial colonization include the oral cavity, skin, respiratory tract, urogenital tract, eyes, and gastrointestinal tract (Cryan et al., 2019). Among these sites, the gut harbors the largest microbial population (Yuan et al., 2021). The gut microbiota comprises yeasts, archaea, parasites, viruses, and protozoa, with bacterial populations being the most predominant (Honarpisheh et al., 2022). These microorganisms play a crucial role in regulating host inflammatory immunity (Levy et al., 2017) and promoting intestinal barrier function (Nie et al., 2021), thereby serving as vital contributors to the maintenance of human health.

The diversity of microbial species increases from childhood to adulthood and subsequently declines in old age (Hou et al., 2022). In healthy adults, the fecal microbiota is predominantly composed of *Proteobacteria*, *Bacillota*, *Actinobacteria*, and *Bacteroidota*, which collectively account for over 90% of the gut microbiome, while the abundance of *Fusobacteria* and *Verrucomicrobia* remains relatively low. The reduction in anaerobic bacteria and *Bifidobacteria* is associated with the worsening of inflammatory conditions, as these bacteria play a crucial role in stimulating the immune system (Rinninella et al., 2019). With the increase in the size of cerebral infarction, *Bacteroidota* tend to overproliferate, whereas *Bacillota* and *Actinobacteria* show a decreasing trend. Certain members of *Bacteroidota*, such as the genus *Bacteroides*, may exacerbate neuroinflammation by promoting the polarization of pro-inflammatory Th17 cells. *Bacillota* include various genera that produce SCFAs, such as *Lactobacillus*, while *Bifidobacterium* within *Actinobacteria* exhibits immunomodulatory functions. A reduction in their populations may lead to weakened anti-inflammatory signals and compromised integrity of the intestinal barrier (Singh et al., 2016a). Disruption of gut microbiota diversity can adversely affect the immune system and increase intestinal permeability, resulting in enhanced infiltration of peripheral immune cells, including T cells and monocytes, into the brain following a stroke. This infiltration further amplifies neuroinflammatory responses and exacerbates brain tissue damage (Ma et al., 2024a).

#### 2.2.2 Gut microbiota-related metabolites influence neuroinflammation

TMAO is a metabolite produced by gut microbiota, primarily derived from dietary choline and L-carnitine through the action of intestinal microorganisms. It is transported to the liver via the portal vein, where it is oxidized into TMAO by flavin monooxygenase 3. The impact of TMAO on neuroinflammation occurs through multiple pathways. On one hand, TMAO may exacerbate neuroinflammation by influencing the activation state of microglia and the phenotypic transformation of monocytes and astrocytes, rendering them more susceptible to a pro-inflammatory phenotype, which leads to the secretion of elevated levels of inflammatory mediators such as IL-6 and MMP-9 (Haghikia et al., 2018; Su et al., 2021; Tu and Xia, 2024). On the other hand, TMAO may exacerbate neuroinflammation by increasing oxidative stress. TMAO inhibits mitochondrial function and activates oxidase systems, which leads to increased electron leakage and the production of superoxide anions. This process further promotes the generation of reactive oxygen species (ROS) (Ke et al., 2018; Chen et al., 2019). These ROS can damage intracellular macromolecules, including lipids, proteins, and DNA, thereby triggering cellular inflammatory responses. Furthermore, TMAO may influence the activity of antioxidant enzymes, such as superoxide dismutase, catalase, and glutathione peroxidase (Li et al., 2017; Brunt et al., 2020). Such influence can weaken the cellular antioxidant defense capacity, leading to an additional increase in ROS levels and exacerbating oxidative stress and neuroinflammation. Additionally, TMAO levels are positively correlated with the expression of various pro-inflammatory cytokines, such as IL-6 and TNF-α, which play a crucial role in post-stroke neuroinflammation. Their increase can further activate immune cells, amplify the inflammatory response, and result in aggravated brain tissue damage (Zhu et al., 2021).

#### 2.2.3 The impact of intestinal gram-negative bacterial outer membrane endotoxins on neuroinflammation

Lipopolysaccharide (LPS), a component of the outer membrane of Gram-negative bacteria, is commonly referred to as an endotoxin. In healthy individuals, the gut microbiota serves as the primary source of LPS in the human body. Following a stroke, LPS can enter the bloodstream by disrupting intestinal barrier function, subsequently activating the body’s immune system. It possesses the ability to cross the blood-brain barrier (BBB) and bind to Toll-like receptor 4 (TLR4) on the surfaces of microglia and astrocytes in the brain. This interaction triggers a cascade of signaling pathways, including the nuclear factor-κB (NF-κB) pathway, which leads to the production of pro-inflammatory cytokines, activation of the innate immune response, and further exacerbation of the inflammatory response within the central nervous system (Fenton and Golenbock, 1998; Chidambaram et al., 2022). Nuclear factor erythroid 2-related factor 2 (Nrf2) is a pivotal transcription factor that serves as the guardian of redox homeostasis and is considered a promising therapeutic target for the treatment of stroke and inflammation-related diseases (Zhang et al., 2017). LPS can diminish the nuclear accumulation of Nrf2, leading to a decrease in its DNA-binding activity and a subsequent reduction in the expression of antioxidant enzymes, such as heme oxygenase-1 and NAD(P)H: quinone oxidoreductase 1. This decline results in a diminished antioxidant capacity in cells, which further exacerbates LPS-induced oxidative stress and inflammatory responses (Liao et al., 2020; Yang and Chen, 2021). Furthermore, LPS induces the production of ROS in microglia. ROS not only directly causes cellular damage but also triggers the activation of both brain-resident (microglia) and peripheral (leukocyte) immune pathways, leading to the release of additional inflammatory mediators and effector molecules. This inflammatory response may impair the reuptake of the neurotransmitter glutamate, resulting in elevated concentrations of glutamate in the synaptic cleft. Such elevations can lead to excitotoxicity, which damages neuronal structure and function, thereby creating a vicious cycle (Liao et al., 2020; Xin et al., 2023).

## 3 MGBA and neuroinflammation

### 3.1 Tools for intestinal microbiome analysis

The emergence of conservative 16S ribosomal RNA (16SrRNA or 16S) high-throughput gene sequencing has enabled the detection and determination of the composition of intestinal flora. While metagenomic sequencing encompasses all the analytical capabilities of 16S sequencing, it also allows for the analysis of bacterial community structure at the species level. However, metagenomic sequencing presents challenges such as higher costs, accuracy that depends on the quality of assembly, and significant influence from host contamination. The 16S rRNA gene is frequently employed in the analysis of intestinal microbial components, with the V3-V4 region being the primary focus of sequencing. This region is utilized to investigate the structure of bacterial communities at levels above the genus, as well as for species composition and diversity analyses. The principal functions involve conducting 16S-α diversity analysis, which assesses the diversity of species within individual samples, and 16S-β diversity analysis, which evaluates the diversity of species between different samples. Both analyses are influenced by environmental factors (Finotello et al., 2016; Willis, 2019).

### 3.2 MGBA

**Figure 1:**
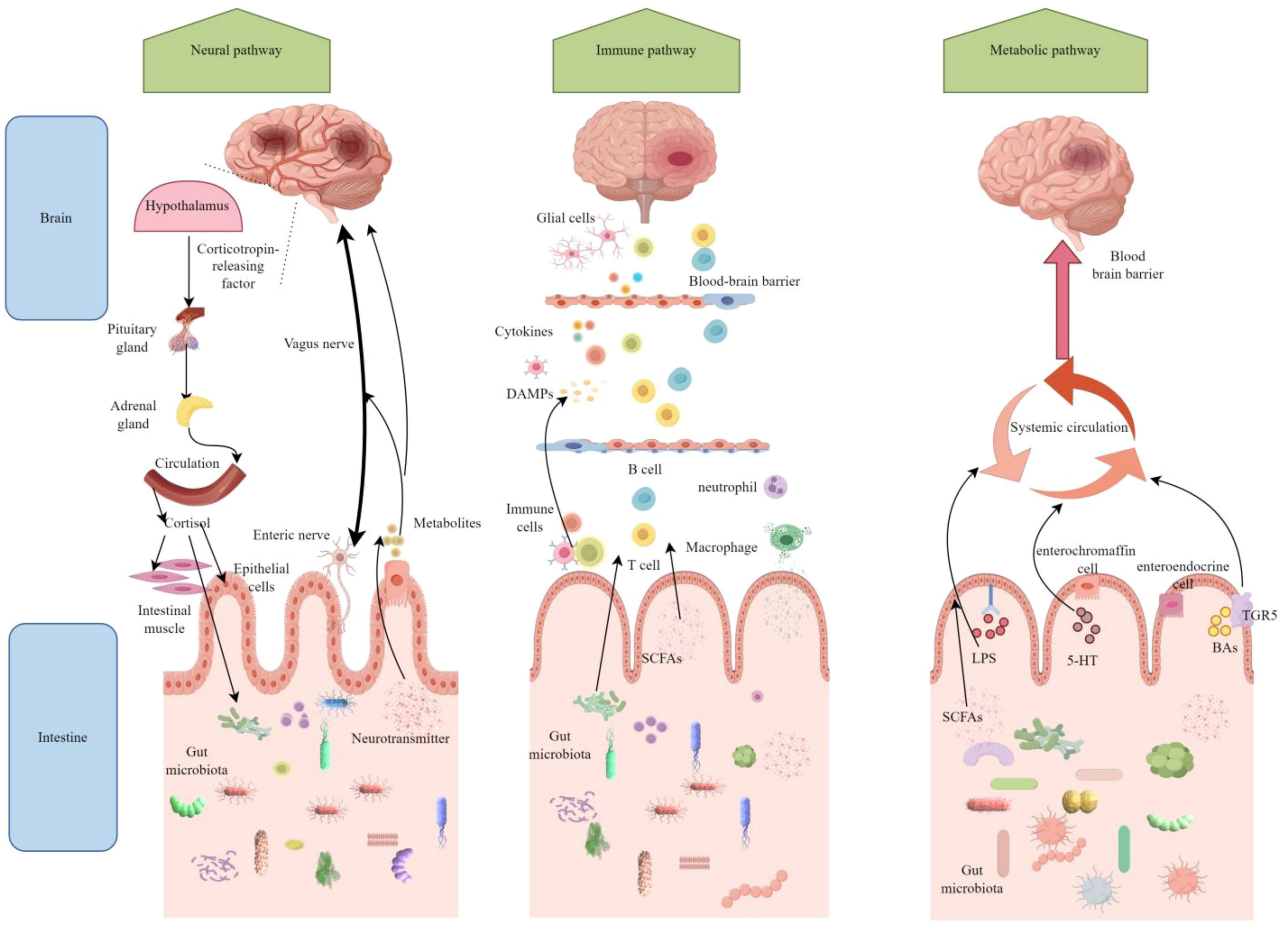
After ischemic stroke, the microbiota-gut-brain axis (MGBA) establishes bidirectional communication through neural, immune, and metabolic pathways. In the neural pathway, cerebral ischemia activates the hypothalamic-pituitary-adrenal (HPA) axis and the vagus nerve, leading to intestinal barrier disruption and dysbiosis. Conversely, the gut microbiota influences brain function recovery through enteric nerve and vagus nerve signaling. Regarding the immune pathway, brain injury triggers systemic inflammation, with gut immune cells, such as T cells, migrating to the brain and exacerbating neuroinflammation. Meanwhile, metabolites produced by the gut microbiota aggravate brain damage by activating microglia. Short-chain fatty acids (SCFAs) produced by the gut microbiota exert neuroprotective effects by crossing the blood-brain barrier; however, harmful metabolites promote stroke progression. Figure generated by Figdraw.(be found in 3.2 MGBA).

The study of interactions between gut microbiota and non-gastrointestinal organs has recently emerged as a significant research focus. Numerous studies have demonstrated bidirectional communication between gut microbiota and the brain, a complex physiological pathway known as the MGBA (Longo et al., 2023). This bidirectional communication between the MGBA and the brain primarily occurs through neural, immune, and metabolic pathways (**Figure 1**).

#### 3.2.1 Neural pathway

The neural pathways illustrate how the brain influences intestinal function through both direct and indirect routes, with the autonomic nervous system (ANS) serving as the primary conduit for the complex bidirectional communication of the MGBA, encompassing both positive and negative feedback mechanisms. The ANS is divided into two components: the sympathetic nervous system (SNS) and the parasympathetic nervous system (PNS). The SNS plays a crucial role in regulating the integrity, permeability, and immune response of the intestinal barrier by modulating the secretion of intestinal mucus, the expression of tight junction proteins in epithelial cells, and the recruitment and activation of immune cells (Tsukamoto et al., 2007; Mallesh et al., 2022; Ten Hove et al., 2023).

The vagus nerve (VN) is a major component of the PNS, regulating intestinal functions, the composition and activity of the microbiota, and facilitating bidirectional communication between the gut and the brain. It plays a central role in the neural pathways of the gut-brain axis (Fülling et al., 2019). The VN transmits information between the brain and the gut through various mechanisms. On one hand, it conveys signals from the brain to the gut via efferent nerve fibers, regulating intestinal motility, secretion, and sensation. On the other hand, it detects metabolites produced by gut microbiota and hormones released by enteroendocrine cells through afferent nerve fibers, transmitting these signals to the central nervous system, thereby influencing autonomic neural integration and adaptive responses in the brain. Following a stroke, damage or dysfunction of the vagus nerve may disrupt the bidirectional communication network between the gut and the brain. The experiment demonstrated that common carotid artery occlusion and vagotomy alter intestinal morphology and microbiome composition, resulting in an increased abundance of *Bifidobacterium* while decreasing the abundance of *Akkermansia* and *Ruminococcus*. Concurrently, these interventions were associated with increased hippocampal neuronal cell death and a higher severity score of neurological functions, alongside changes in hippocampal lipid profiles, elevated lipid peroxidation, and altered expression of inflammatory cytokines. These findings suggest that the vagus nerve plays a crucial role in modulating neuroinflammation, neuronal damage, and the dynamic equilibrium of the gut microbiome following a stroke (Zhang et al., 2024). Electroacupuncture stimulation of the left vagus nerve in rats with cerebral infarction reperfusion significantly increased both the total movement distance and average speed in the open field test. Furthermore, it reduced Evans blue leakage and the fluorescence intensity of FITC-dextran, decreased microglial polarization, and improved the infiltration of inflammatory cells while preserving crypts in colon tissue, resulting in more intact tight junctions between colon cells (Wang et al., 2024). This study indicates that vagus nerve stimulation can influence pathological processes such as neuroinflammation, blood-brain barrier disruption, and intestinal barrier damage in the early stages following a stroke. Consequently, it provides an opportunity for early intervention that may mitigate acute injury from stroke, reduce disability rates, and lower mortality. Current clinical studies have confirmed the enhancing effect of VN on functional recovery following a stroke. This is evidenced by a randomized controlled trial that examined the efficacy of VNS combined with rehabilitation for upper limb motor function in patients with ischemic stroke (Dawson et al., 2021). The integration of vagus nerve stimulation with existing stroke treatment modalities, such as pharmacotherapy and rehabilitation training, results in a synergistic effect that promotes neurological recovery and enhances the quality of life for patients.

Additionally, stroke stress activates the hypothalamic-pituitary-adrenal (HPA) axis, resulting in the release of corticotropin-releasing hormone and cortisol. This process alters intestinal permeability and the composition of gut microbiota. Conversely, gut microbiota metabolites, such as tryptophan derivatives, can feedback to regulate HPA axis activity (Zhou et al., 2023). Within the gut-brain axis, cortisol modulates the activity of serotonin synthase in intestinal neurons, thereby influencing serotonin levels in the gut. Serotonin, in turn, can feedback to regulate HPA axis activity, creating a complex network of interactions.

#### 3.2.2 Immune pathway

The immune pathway represents a crucial mechanism through which the MGBA connects the gut microbiota to the brain. This pathway primarily regulates the activation of gut immune cells, the migration of these cells across systems, and the production of microbial metabolites. Following a stroke, immune cells, including dendritic cells and macrophages, detect pathogens and damage signals within the gut, thereby activating local immune responses through the secretion of pro-inflammatory cytokines, such as IL-6, IL-1β, and TNF-α (Honarpisheh et al., 2022). Additionally, these cells can migrate to the mesenteric lymph nodes and other immune organs, where they activate T cells and B cells to elicit specific immune responses (Cheng et al., 2023). γδ T cells and neutrophils within the gut have the capacity to migrate to the brain, thereby exacerbating inflammatory damage in this organ (Brea et al., 2021). Furthermore, alterations in gut microbiota and their metabolites may influence the differentiation and functionality of immune cells. For instance, SCFAs can inhibit the activity of histone deacetylases, enhance the expression of anti-inflammatory cytokines, and suppress the production of pro-inflammatory cytokines, thereby exerting immunomodulatory effects (Rooks and Garrett, 2016).

#### 3.2.3 Metabolic pathway

Metabolic pathways play a central role in the MGBA by generating microbial metabolites and regulating host physiological functions. These pathways significantly impact neural signal transmission, immune regulation, and energy homeostasis. They primarily operate through the systemic circulation of bioactive metabolites, cerebrospinal fluid circulation, the lymphatic system, and the increased permeability of the BBB. In terms of the circulation of metabolic products in body fluids, metabolites produced by gut microbiota, such as SCFAs, bile acids, and tryptophan metabolites, can enter the brain via the bloodstream and exert direct effects on brain cells. For instance, SCFAs can bind to G protein-coupled receptors in the brain, inhibiting the production of pro-inflammatory cytokines (Kimura et al., 2020). Furthermore, cerebrospinal fluid can exchange substances with the intestines through the lymphatic system, transporting the metabolic products of gut microbiota and inflammatory signals into the brain. Following a stroke, this function becomes impaired, negatively impacting substance exchange and immune regulation between the gut and the brain (Mestre et al., 2020; Chen et al., 2021; Shlobin et al., 2024). Besides, following a stroke, the permeability of the BBB increases, facilitating the entry of microbial metabolites, cytokines, and immune cells from the bloodstream into the brain parenchyma. Concurrently, the disruption of the BBB impairs normal communication between the brain and the gut, thereby disturbing the balance of the brain-gut axis (Wang et al., 2024).

### 3.3 Intestinal microbiota and stroke

Pathological alterations in gut microbial composition, leading to changes in intestinal and neuroimmune status, are collectively referred to as gut dysbiosis. This condition is characterized by several core features, including alterations in microbial composition (e.g., an imbalance in the *Bacillota*/*Bacteroidota* ratio), abnormal metabolic functions (e.g., reduced levels of SCFAs), and dysregulated host-microbiota interactions (e.g., loss of immune tolerance) (Chidambaram et al., 2022).

#### 3.3.1 Intestinal dysbiosis exists in experimental IS

All experimental models of ischemic stroke studied to date, including the intraluminal suture middle cerebral artery occlusion model, photochemically induced model, thromboembolic model, endothelin-1 induced model, embolic microsphere model, and genetically engineered models, have consistently demonstrated alterations in gut microbiota (Kaneko et al., 1985; Miyake et al., 1993; Chen et al., 2015; Sommer, 2017; Kuo et al., 2021; Tuo et al., 2022). Significant differences in gut microbiota exist between young and aged mice, with the *Bacillota* to *Bacteroidota* ratio being approximately nine times higher in aged mice compared to their younger counterparts. Following MCAO in mice, the gut microbiota of young mice resembles that of uninjured aged mice, evidenced by a more than threefold increase in the *Bacillota* to *Bacteroidota* ratio, alongside a reduction in the expression of intestinal tight junction protein and elevated plasma endotoxin levels. In aged mice, the *Bacillota* to *Bacteroidota* ratio increases by approximately 40%, accompanied by a marked decrease in the abundance of *Akkermansia* and an increase in conditional pathogens, such as *Proteobacteria* (e.g., *Enterobacteriaceae*) (Spychala et al., 2018). This indicates that stroke itself exacerbates dysbiosis of the gut microbiota, irrespective of the host’s age. Following a stroke, the abundance of beneficial bacteria, such as *Bifidobacterium* and *Lactobacillus*, significantly decreases, while the quantity of harmful bacteria associated with inflammatory responses and immune dysregulation, including *Proteobacteria* and *Enterobacteriaceae*, markedly increases. After a stroke, intestinal barrier function is compromised, and the levels of SCFAs are significantly reduced, leading to the translocation of endotoxins (e.g., LPS) into the bloodstream. This process may exacerbate intestinal inflammation and subsequently trigger systemic inflammatory responses. The use of antibiotics significantly impacts gut microbiota following a stroke. The widespread administration of antibiotics markedly reduces the diversity of gut microbiota, leading to a decline in the abundance of beneficial bacteria, including *Lactobacillus* and *Ruminococcus*. In contrast, conditionally pathogenic bacteria, such as *Bacteroides*, are not effectively suppressed (Wang et al., 2022). Relevant studies indicate that antibiotic-induced dysbiosis may persist for several weeks, impairing the normal functioning of the MGBA and delaying recovery post-stroke (Spychala et al., 2018). Conversely, other studies have reported that antibiotic treatment, whether administered alone or in combination, can reduce the volume of cerebral infarction (Benakis et al., 2020), as detailed in section 4.4.

#### 3.3.2 The gut microbiota of IS subjects has undergone alterations

To further understand the differences in gut microbiota between stroke patients and healthy controls, we identified the 15 most recent clinical studies that compare the microbiota of stroke patients with that of healthy controls (**Table 1**). The selection of these articles was guided by the PRISMA statement, which assesses the quality of the included literature. Detailed search strategies and flow diagrams used in each database are provided in the supplementary materials.

**Table 1:** Major gut microbiota changings in clinical stroke (be found in 3.3.2 The gut microbiota of IS subjects has undergone alterations).

Type of stroke	Cohort	background	Microbiome method	α-diversity in stroke	Gut microbiota changes	Reference
IS	47IS(①NIHSS ≤ 4 points,16 patients;②NIHSS 5–15 points,21 patients;③NIHSS≥16 points,10 patients)and 15 controls	Chinese	16SrRNA	NIHSS≥16 points:Shannon↓	NIHSS ≤ 4 points, *Lactobacillales order*, *Firmicutes phylum*, *Klebsiella genus*↑;*Escherichia-Shigella genus*, *Bacteroidales order*, *Bacteroidota phylum*↓. NIHSS5–15 points, *Klebsiella genus, Verrucomicrobiota phylum, Verrucomicrobiales order*↑;*Bacteroidales order,Bacteroidota phylum,Bacteroidia,Escherichia-Shigella genus*↓. NIHSS≥16 points, *Actinobacteriota phylum,Klebsiella genus,Coriobacteriaceae family, Collinsella genus*↑;*Escherichia-Shigella genus*, *Bacteroidales order*, *Bacteroidota phylum*↓.	(Bao et al., 2024)
IS	LVO 63 patients, and CSVD 64 patients, 36 controls	Chinese	16SrRNA	Observed species,Chao1,ACE↑	*Bifidobacterium, Butyricimonas, Bifidobacterium longum*↑; *Blautia, Dorea*↓.	(He et al., 2024a)
IS	LCI 65 patients and 65 controls	Chinese	16SrRNA	No difference in Simpson and Shannon	*Lactobacillus, Streptococcus*, *Veillonella, Acidaminococcus*↑;*Agathobacter, Lachnospiraceae_UCG-004*↓.	(Ma et al., 2024b)
IS and HS	IS 20 patients,HS 15 patients and 35 controls	Chinese	16SrRNA	Shannon↑	IS: *Butyricimonas,Alloprevotella,Escherichia*↑;*Roseburia, Streptococcus salivarius*↓. HS: *Atopobium,Hungatella,Eisenbergiella*, *Butyricimonas,Odoribacter,Lachnociostridium, Alistipes,Parabacteroides*, *Fusobacterium*↑; *Ruminococcus,Coprococcus*↓.	(Chen et al., 2024b)
IS	LAA 61 patients,CE 20 patients and 51 controls	Chinese	16SrRNA	Shannon↑	LAA: *Firmicutes*, *Proteobacteria*, *Actinobacteria*, *Parabacteroides, Klebsiella, Desulfovibrio, Enterococcus, Eubacterium, Lactobacillus, Bifidobacterium*↑;*Bacteroidetes*↓.	(Xu et al., 2021)
IS	IS 10 patients and 21 controls	Italy	16SrRNA	Chao1↓	*Verrucomicrobia,Proteobacteria phylum, Verrucomicrobiaceae,Bacteroidaceae, Phascolarctobacterium,Alistipes,Christensenellaceae,Sutterella*↑;*Actinobacteria, Clostridiaceae, Ruminococcaceae,Coriobacteriaceae, Anaerostipes,Clostridiales, Akkermansia,Bacteroides*↓.	(Marsiglia et al., 2023)
IS	IS 30 patients and 30 controls	Chinese	Metagenomic sequencing	Observe,Chao1,Ace, Shannon↑	*Oscillibacter, Clostridium, Odoribacter, Akkermansia, Ruminococcus*↑.	(Zhao et al., 2022)
IS	IS 14 patients and 17 controls	Chinese	16SrRNA	No difference in Chao1 and Shannon indices	SS(subacute stroke) vs controls: *actobacillaceae*↑; *Butyricimonas,Peptostreptococcaceae,Romboutsia,Anaerotruncus,Blautia_massiliensis,Fusicatenibacter*↓. CS(convalescent stroke) vs controls: *Lactobacillaceae,Aerococcaceae,Abiotrophia,Eubacterium_siraeum*↑, *Butyricimonas,Peptostreptococcaceae,Romboutsia,Escherichia,Marinifilaceae*↓. SS vs CS: SS, *Veillonella, Lactobacillus_fermentum*↑.	(Cui et al., 2023)
AIS	AIS 95 patients and 81 controls	Chinese	16SrRNA	Chao1↑and ACE↑	*Parabacteroides*, *Alistipes*↑; *Prevotella*, *Roseburia*↓	(Li et al., 2025)
AIS	(SS)NIHSS ≤ 14 point, 33 patients and (MS)NIHSS>15 point, 32 patients	Brazil	16SrRNA	SS: Pielou↓	SS: *Pseudomonadota phylum*, *Campylobacter genus*, *Finegoldi genus*, *Pseudobutyrucucoccus genus*↑; MS:*Gemmiger genus, Bilophila genus, Bifidobacterium genus*↑.	(Pilon et al., 2025)
AIS	140IS(①NIHSS ≤ 4 points,78 patients;②NIHSS5–15 points,47 patients;③NIHSS≥16 points,15 patients)and 92 controls	Chinese	16SrRNA	No difference in Chao1 and Shannon indices	IS: *Lactobacillaceae,Akkermansia,Enterobacteriaceae,Porphyromonadaceae*↑;*Roseburia,Bacteroides,Lachnospiraceae,Faecalibacterium,Blautia,Anaerostipes*↓.	(Tan et al., 2021)
AIS	(HS 18 patients;IS 7 patients) and 26 controls	South Korea	16SrRNA	Shannon↓	*Parabacteroides*, *Escherichia_Shigell*, *Lachnoclostridium*, *Fusobacterium*,*Lactobacillales*,*Enterococcus*↑;*Prevotella*,*Faecalibacterium*,*Prevotella,Faecalibacterium,Roseburia,Selenomonadales,Lachnospiraceae_NK4A136_group,Eubacterium,Dialister*↓.	(Park et al., 2023)
IS	IS 79 patients and 98 controls	Chinese	16SrRNA	No difference in Chao1 and Shannon indices	IS: *Lactobacillus, Lactococcus, Actinobacteria, Proteobacteria*↑; *Faecalibacterium, Subdoligranulum, Eubacterium rectal group, Roseburia, Lachnoclostridium, Butyricicoccus*↓.	(Li et al., 2020)
IS	CS 30 patients and 27 controls	Chinese	16SrRNA	Observed_species↑,Chao1↑,Shanno↑,ACE↑	CS: Enterobacteriaceae, Streptococcaceae, Lactobacillaceae, Escherichia–Shigella, Streptococcus, Lactobacillus, Klebsiella↑; Veillonellaceae, Faecalibacterium, Dialister, Roseburia↓.	(Zheng et al., 2022)
IS	(IS 287 patients, TIA 25 patients, HS 37 patients)and 51 controls	The Netherlands	16SrRNA	Shannon,Inverse Simpson,Observed Taxa↓	IS, HS: *Proteobacteria, Escherichia/Shigella, Peptoniphilus, Ezakiella, Enterococcus*↑; *Firmicutes, Bacteroidetes, Blautia, Subdoligranulum, Bacteroides*↓.	(Haak et al., 2021)

IS, Ischemic Stroke; AIS, Acute ischemic stroke; MS, mild/moderate stroke; SS, severe stroke; CI: cerebral infarction; CS, cryptogenic stroke; LVO, large vessel occlusion; CSVD, cerebral small vessel disease; LCI, Lacunar Cerebral Infarction; HS, hemorrhagic stroke; LAA, large artery atherosclerotic stroke; CE, cardioembolic stroke; TIA, transient ischemic attack.

(1) Most studies utilized 16S rRNA gene sequencing, while a minority employed metagenomic sequencing, focusing exclusively on the bacterial components of the gut microbiota. (2) The research involved teams from various countries and regions, including China, Italy, Brazil, South Korea, and the Netherlands, reflecting a global interest in the relationship between gut microbiota and stroke. Dietary habits, environmental factors, and other variables across different regions may influence both the gut microbiota and the incidence of stroke.(3)Each study employed distinct grouping methods tailored to the research objectives and the specific conditions of the patients. Some studies categorized patients based on the type of stroke, such as large artery atherosclerosis or cardioembolic stroke, while others classified them according to the severity of the condition, including mild, moderate, and severe stroke. Additionally, some studies considered coagulation function, differentiating between hypercoagulable states and normal coagulation function, or the time of onset, distinguishing between the subacute phase and the recovery phase. Furthermore, certain studies concentrated on specific types of stroke, including cryptogenic stroke and lacunar infarction. These varied grouping approaches enable a more nuanced investigation of the role of gut microbiota across different types and stages of stroke. (4)Conversely, the differences in α-diversity between stroke patients and healthy controls exhibited variability across studies. This inconsistency may be attributable to several factors, including the characteristics of the study subjects, sample size, study design, and the type and severity of stroke.

Overall, *Parabacteroides* has been referenced in numerous studies, demonstrating a significant increase in abundance among patients with AIS and those suffering from large artery atherosclerosis (LAA) stroke. Similarly, *Alistipes* has also shown increased abundance in research involving AIS and IS patients. *Streptococcus* is more abundant in the gut microbiota of patients with IS and cryptogenic stroke (CS). *Klebsiella* exhibits an increasing trend in abundance among patients with LAA-type stroke and some patients with AIS. *Lactobacillus* shows increased abundance in stroke patients, particularly in those with specific types of stroke, such as lacunar infarction, or during particular stages, such as the recovery phase. The abundance of *Escherichia-Shigella* is elevated in patients with CS and some individuals with IS. Research has demonstrated that the prevalence of *Enterobacteriaceae* increases in patients diagnosed with CS. Conversely, the abundance of *Prevotella* significantly declines in patients with AIS, particularly those with LAA type stroke, as well as in individuals with IS. The abundance of *Bacteroides* is reduced in the gut microbiota of patients with AIS and CS. *Faecalibacterium* shows a declining trend in patients with CS and some individuals with IS. Moreover, *Roseburia* displays a reduced abundance in stroke patients, including those with AIS. Research has also indicated a decrease in the abundance of *Clostridiales* among patients with IS. Additionally, *Lachnospiraceae* exhibits decreased abundance in stroke patients, particularly among certain AIS patients. Finally, *Akkermansia* is found to be less abundant in specific stroke patients, including those with IS.

## 4 The effects of different therapeutic measures on IS neuroinflammation

### 4.1 Gut microbiota-related metabolites

The primary metabolic byproduct of a high-fiber diet in the colon is SCFAs (Fusco et al., 2023). SCFAs serve as a crucial energy source for both the microbiota and the endothelial cells of the gut, playing a significant role in maintaining intestinal immune balance and regulating the polarization and induction of T cells (Sadler et al., 2020). The maturation, function, and maintenance of homeostasis in microglial cells are dependent on SCFAs (Liu et al., 2023). Gut microbial communities that produce SCFAs are essential for sustaining intestinal homeostasis, strengthening the intestinal epithelial mucus layer, regulating oxidative stress, and preventing the entry of harmful pathogens (Colombo et al., 2021).

Bile acids (BAs) are endogenous metabolites produced by both the host and microorganisms, playing a critical role in determining the abundance, diversity, and metabolic activity of microbial communities (Larabi et al., 2023). BAs influence microbiota composition through their antimicrobial properties and the activation of host signaling pathways that maintain intestinal homeostasis. The activation of the BA receptor TGR5 reduces the levels of cleaved caspase-8 and NLRP3, leading to a decrease in inflammatory factor accumulation and alleviation of neuroinflammation (Liang et al., 2021a). Furthermore, BAs are essential for maintaining a healthy gut microbiota and supporting innate immunity (Collins et al., 2023).

TMAO is primarily formed through the metabolism of red meat, eggs, and fish in the human body, and it has been demonstrated to promote atherosclerosis, thrombosis, and inflammation (Simó and García-Cañas, 2020). Numerous studies have indicated that elevated levels of TMAO can increase the area of cerebral infarction and are negatively correlated with post-stroke recovery (Su et al., 2021; Zhu et al., 2021). Furthermore, TMAO may serve as a biomarker for predicting the risk of stroke recurrence, potentially providing new avenues for future treatment strategies (Xu et al., 2022).

**Figure 2:**
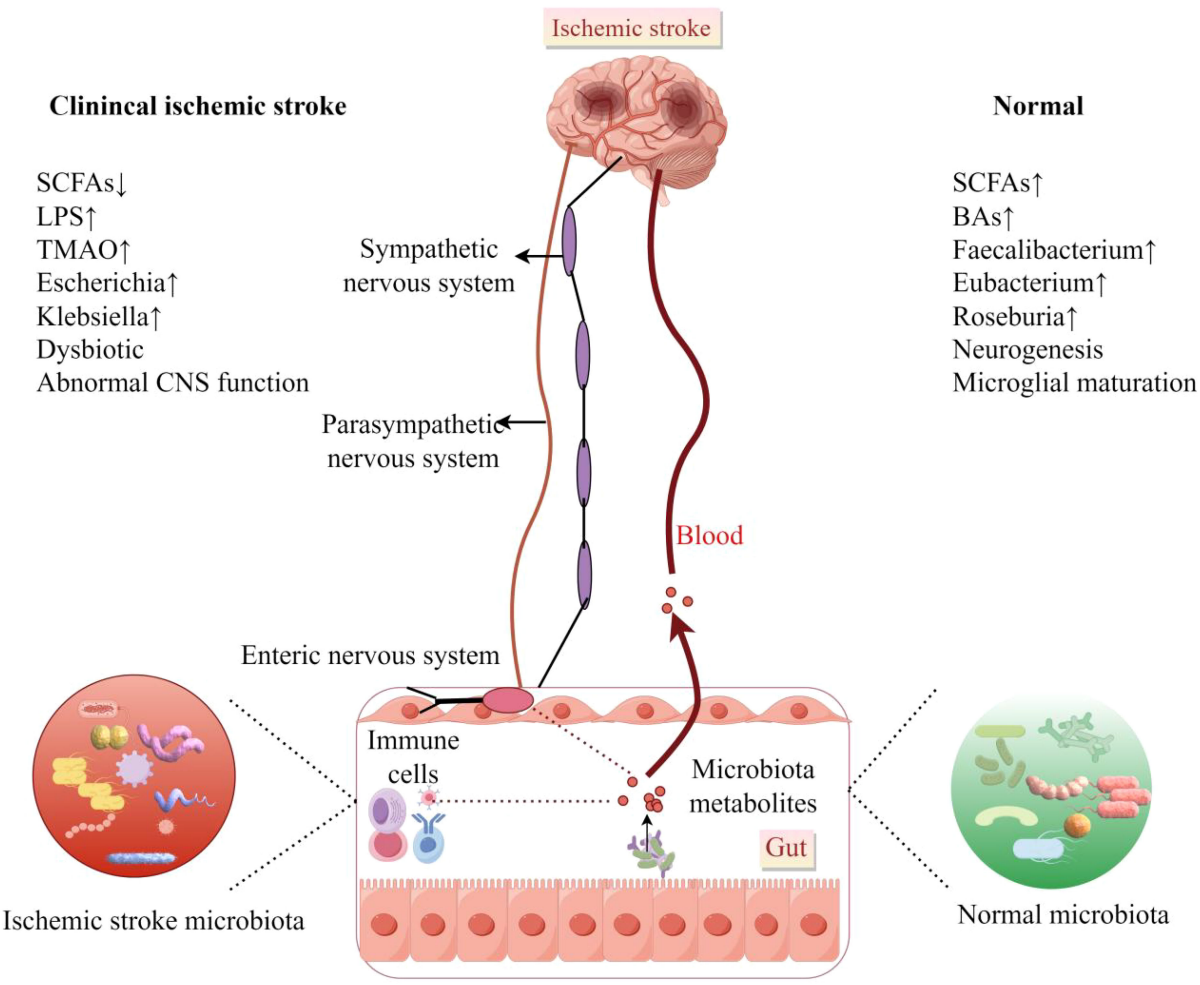
There are notable differences in gut microbiota and their metabolites between healthy individuals and stroke patients. However, diet can modulate these microbiota and their metabolites, emerging as an adjunctive therapeutic strategy for managing neuroinflammation following a stroke. Generally, foods rich in choline and phosphocholine are associated with poorer stroke outcomes, whereas those high in resistant starch and fiber have been shown to improve stroke outcomes. Figure generated by Figdraw.((be found in 4.1 Gut Microbiota-Related Metabolites).

In summary, the modulation of gut microbiota metabolites through dietary interventions—specifically, the enhancement of SCFAs, the reduction of TMAO, and the maintenance of bile acid equilibrium—may represent an adjunctive therapeutic strategy for addressing post-stroke neuroinflammation. This approach requires individualized nutritional interventions and sustained metabolic monitoring. **Figure 2**.

### 4.2 Prebiotics and probiotics

Probiotics, defined as live microbial strains, and prebiotics, which are non-digestible food components that promote the growth and/or activity of beneficial microorganisms, play a significant role in modulating gut microbiota and immune responses, thereby maintaining human health. (1)In regulating microbial balance, probiotics, such as *Lactobacillus* and *Bifidobacterium*, directly supplement beneficial bacteria, inhibit the growth of pathogenic bacteria, and restore the balance of gut microbiota. Research has demonstrated that the combined supplementation of *Limosilactobacillus reuteri* GMNL-89 and *Lacticaseibacillus paracasei* GMNL-133 can reduce infarct volume in stroke mice and enhance intestinal barrier integrity (Wang et al., 2025b). Prebiotics, including inulin and fructooligosaccharides, indirectly increase the production of SCFAs by selectively promoting the proliferation of beneficial bacteria (Romo-Araiza and Ibarra, 2020; Rahman et al., 2024). SCFAs, such as butyrate, are known to upregulate brain-derived neurotrophic factor, alleviate neuroinflammation, and promote synaptic plasticity (Cruz-Martínez et al., 2024).(2)Probiotics play a significant role in mitigating neuroinflammation and oxidative stress by decreasing pro-inflammatory factors such as TNF-α and IL-6, while simultaneously increasing anti-inflammatory factors like IL-10 (Zhong et al., 2021; Wang et al., 2023b; Cruz-Martínez et al., 2024). In studies involving stroke rats supplemented with probiotics, a notable reduction in TNF-α levels in the hippocampal region was observed, alongside an increase in BDNF expression (Cruz-Martínez et al., 2024). Additionally, prebiotics contribute to the reduction of ROS accumulation by enhancing the activity of antioxidant enzymes, such as glutathione peroxidase, thus providing protection to neurons against oxidative damage (Al Suhaibani et al., 2021). (3) Furthermore, concerning the protection of the BBB and immunomodulation, probiotics like GMNL-133 have been shown to decrease BBB permeability, preventing the infiltration of peripheral inflammatory cells into brain tissue. They also modulate systemic inflammatory responses by influencing T cell subsets, including the Th1/Th2 balance and cytotoxic T cell activity in the spleen (Agarwal et al., 2025; Wang et al., 2025b).

In clinical research, the combination of probiotics with enteral nutrition has been shown to shorten the hospital stay of stroke patients, improve their nutritional status, and reduce the risk of complications, including pulmonary infections (Zhong et al., 2021). The concurrent administration of prebiotics (e.g., inulin) and probiotics (e.g., Enterococcus faecium) has also been demonstrated to enhance post-stroke spatial memory and learning abilities, a mechanism associated with the upregulation of brain-derived neurotrophic factor (BDNF) (Liu et al., 2020b; Cruz-Martínez et al., 2024). Furthermore, the combined use of probiotics (Bifidobacterium and Lactobacillus) has been found to decrease the incidence of post-stroke depression and lower serum inflammatory markers, such as nuclear factor kappa-light-chain-enhancer of activated B cells (NF-κB) and interleukin-1 beta (IL-1β) (Wang et al., 2023b).

Prebiotics and probiotics present both opportunities and challenges for future therapies. (1) Personalized microbiota interventions, tailored to the patient’s gut microbiota characteristics—such as SCFAs levels and microbiota diversity—may enhance therapeutic efficacy (Liu et al., 2020b). (2) Genetically modified probiotics, such as engineered strains of Escherichia coli Nissle (EcN), can facilitate the targeted delivery of anti-inflammatory molecules (e.g., carbon monoxide and hydrogen sulfide), alleviate oxidative stress, restore a favorable gut microbiota environment, and enhance local therapeutic effects (Ma et al., 2025; Zhang et al., 2025). (3) The development of combination therapy interventions is also a promising trend. For instance, Buyang Huanwu Decoction combined with probiotics may regulate the gut-brain axis through multiple targets (Liang et al., 2021b). Furthermore, the combination of probiotics with antioxidants, such as curcumin, may synergistically inhibit neuroinflammation (Śliwińska and Jeziorek, 2021). (4) However, the specificity and long-term stability of probiotic strains require further investigation, as different strains exhibit significant variations in efficacy, necessitating additional screening.

Prebiotics and probiotics exhibit significant potential for neuroprotection following a stroke by modulating gut microbiota, inhibiting neuroinflammation, and repairing the blood-brain barrier (BBB). Future research should focus on integrating precision medicine and engineering technologies to optimize strain combinations and administration protocols, thereby enhancing their clinical applicability.

### 4.3 FMT

The exacerbation of post-stroke neuroinflammation is closely associated with an imbalance in intestinal microbiota, and FMT can effectively reconstruct a healthy intestinal microecology. FMT alleviates post-stroke neuroinflammation and intestinal microbiota dysbiosis through multiple pathways: (1) regarding the regulation of microbiota composition, FMT increases the abundance of beneficial bacteria (such as *Lactobacillus* and *Prevotella*), reduces opportunistic pathogens (such as *Akkermansia*), restores levels of SCFAs (such as *butyrate*), and enhances intestinal barrier function by upregulating the expression of occludin and mucin-2 (Zhang et al., 2021; Huang et al., 2024). (2) In terms of inhibiting inflammatory pathways, FMT can downregulate the TLR4/NF-κB signaling pathway, reduce the pro-inflammatory polarization of microglia (M1 type), decrease pro-inflammatory factors such as IL-17 and IFN-γ, while increasing the anti-inflammatory factor IL-10 (Hediyal et al., 2024; Li et al., 2024). Regarding neuronal protection, FMT reduces the expression of the apoptotic protein Bax, increases the anti-apoptotic protein Bcl-2, and alleviates ischemic brain injury (Hediyal et al., 2024). Multiple animal experiments have demonstrated that FMT improves post-stroke neurological function. By restoring the balance of gut microbiota, FMT significantly reduces infarct volume, decreases microglial activation, protects the blood-brain barrier, increases striatal levels of 5-HT and DA, enhances motor function, and alleviates depressive-like behaviors (Zhang et al., 2021; Huang et al., 2024; Jiang et al., 2025).

However, there are significant obstacles to clinical translation. First, it is imperative to conduct stringent screening of donors to prevent pathogen transmission and immune rejection, thereby ensuring the safety of recipients (Hediyal et al., 2024). Second, individual differences in microbiota may influence the long-term efficacy of transplantation (Wang et al., 2025a). Additionally, the specific regulatory network of the gut-brain axis requires further elucidation (Yuan et al., 2021). FMT presents a revolutionary therapeutic strategy for addressing post-stroke neuroinflammation by restoring the balance of the gut microbiome. In the future, rigorously designed clinical trials will be necessary to verify its safety and efficacy, as well as to explore standardized transplantation protocols, such as encapsulated FMT. The integration of microbiome studies with artificial intelligence analysis holds promise for achieving precise interventions and advancing stroke rehabilitation therapy.

### 4.4 Antibiotics

The application of antibiotics in experimental stroke models presents significant paradoxes. On one hand, antibiotics may exert neuroprotective effects by inhibiting harmful bacterial translocation and alleviating neuroinflammation. On the other hand, antibiotic-induced dysbiosis of the gut microbiota may exacerbate post-stroke injury and hinder functional recovery. This paradox arises from the bidirectional regulatory mechanisms of the gut-brain axis and the non-selective effects of antibiotics on the microbiome.

In terms of neuroprotective mechanisms, antibiotics mitigate systemic inflammatory responses following a stroke by inhibiting the excessive proliferation of pathogenic bacteria and reducing the risk of microbial translocation (Sun et al., 2022; Chen et al., 2024a). For instance, in stroke patients with concurrent COVID-19 infection, the enrichment of *Enterobacteriaceae* is significantly associated with poor prognosis, and early antibiotic intervention may improve outcomes (Chen et al., 2024a). Regarding the regulation of immune cell dynamics, the immunosuppressive state post-stroke is prone to trigger intestinal infections. Antibiotics can indirectly reduce the depletion of peripheral immune cells (e.g., splenic lymphocyte reduction) by controlling infections, thereby maintaining immune homeostasis (Pu et al., 2024). Furthermore, antibiotic pretreatment can reduce microglial activation and decrease the release of pro-inflammatory factors (TNF-α, IL-1β) (Wei et al., 2024). Additionally, antibiotics can enhance vascular endothelial function by reducing harmful metabolites elevated post-stroke (e.g., TMAO, PAGln) and by targeting the inhibition of TMAO-producing microbiota (Zhang et al., 2023; Qu et al., 2024).

However, antibiotics can disrupt the microbiota responsible for the production of SCFAs. They non-specifically eliminate beneficial bacteria, including those that produce SCFAs like *Ruminococcaceae* and *Faecalibacterium*, resulting in decreased SCFA levels. SCFAs, particularly butyrate, exhibit anti-inflammatory properties, help maintain the integrity of the blood-brain barrier, and promote synaptic plasticity. A reduction in SCFAs is associated with the exacerbation of neurological deficits following a stroke (Sun et al., 2022; Zhang et al., 2023; Qu et al., 2024). Conversely, prolonged antibiotic use may further damage the intestinal mucosal structure, increasing the risk of ‘leaky gut’ and facilitating the entry of endotoxins into the bloodstream (Jiang et al., 2023; Shen et al., 2025). In stroke models treated with antibiotics, the expression of intestinal tight junction proteins is diminished, exacerbating systemic inflammation (Yang et al., 2024). Additionally, antibiotics may inhibit the metabolic activity of microbes that metabolize drugs (such as antiplatelet agents), and certain antibiotics (such as broad-spectrum β-lactams) may directly interfere with the microbial synthesis pathways of neurotransmitters (such as GABA) (Zhang et al., 2023).

In clinical applications of antibiotic therapy, the precise selection and optimization of antibiotic duration can be achieved by utilizing narrow-spectrum antibiotics that specifically target pathogenically enriched bacteria post-stroke, such as *Enterococcus*. Limiting the duration of antibiotic use to seven days or fewer can significantly reduce the risk of dysbiosis (Wang et al., 2023a; He et al., 2024b). Concurrently, microbiome interventions can be implemented by supplementing with SCFAs producing bacteria, such as *Faecalibacterium*, or dietary fiber following antibiotic treatment to restore microbial balance (Wang et al., 2023a; Xu et al., 2025). Alternatively, FMT can be employed to reconstruct the functional microbiota and enhance stroke outcomes after antibiotic-induced dysbiosis (Pluta et al., 2021). Furthermore, microbiota improvement may be achieved through biomarker-guided individualized treatment by screening subgroups likely to benefit from antibiotic therapy based on the characteristics of the patient’s gut microbiota, such as the *Alistipes/Prevotella* ratio or plasma LPS levels (Chang et al., 2021; Li et al., 2025).

The paradoxical effects of antibiotics in stroke models illustrate the dual nature of the gut microbiota. In future research, it will be essential to balance their neuroprotective benefits against the risk of dysbiosis through targeted intervention strategies, ultimately aiming to enhance stroke prognosis by modulating the microbiome.

## 5 Conclusion

In this article, we examine the relationship between gut microbiota and the pathogenesis of IS induced neuroinflammation. The gut microbial community is intricately linked to post-stroke neuroinflammation. Following an ischemic stroke, dysbiosis of the gut microbiota can result in compromised intestinal barrier function and increased permeability. This condition allows bacterial toxins and metabolites to enter the bloodstream, thereby activating the immune system and triggering a systemic inflammatory response. Consequently, this process affects the brain via the MGBA, exacerbating neuroinflammation. Additionally, neurotransmitters, cytokines, immune mediators, and microbiota-derived metabolites function as signaling molecules within the MGBA, facilitating communication between the gut and the brain and contributing to the development of post-stroke neuroinflammation. Recent findings from preclinical and clinical studies on IS have confirmed a bidirectional regulatory relationship between IS and gut microbiota dysbiosis, establishing the MGBA as a novel therapeutic target for ischemic stroke. The underlying mechanisms involve microbial metabolites, such as SCFAs and BAs, immune modulation, and the neuroendocrine network. Given the pivotal role of the MGBA, current strategies aimed at improving stroke outcomes include modulating the gut microenvironment and intervening in neuroinflammatory responses through approaches such as FMT, the application of probiotics and prebiotics, antibiotics, and dietary interventions. Furthermore, translating preclinical research findings into clinical practice necessitates large-scale, multicenter clinical studies to clarify the applicable population, optimal treatment timing, and dosage, thereby promoting the clinical application and dissemination of related therapeutic strategies.

The MGBA plays a central role in the neuroinflammation associated with ischemic stroke. Intervention strategies are evolving from single anti-inflammatory approaches to multi-target regulation, encompassing microbiota, metabolism, immune response, and neural interactions. Future efforts should emphasize the mechanism-clinical translation loop, integrating precision medicine and interdisciplinary technologies to establish a new paradigm in stroke treatment.
