# Supplementary material for: The regulation of neuroinflammatory response after stroke by intestinal flora microorganisms
Source: Front Cell Infect Microbiol. 2025 Jun 23;15:1594834. doi: 10.3389/fcimb.2025.1594834 (PMC12230021; doi:10.3389/fcimb.2025.1594834)
Supplement: Supplementary file 1 [file Table1.docx]

Supplementary Material

# Supplementary Figures


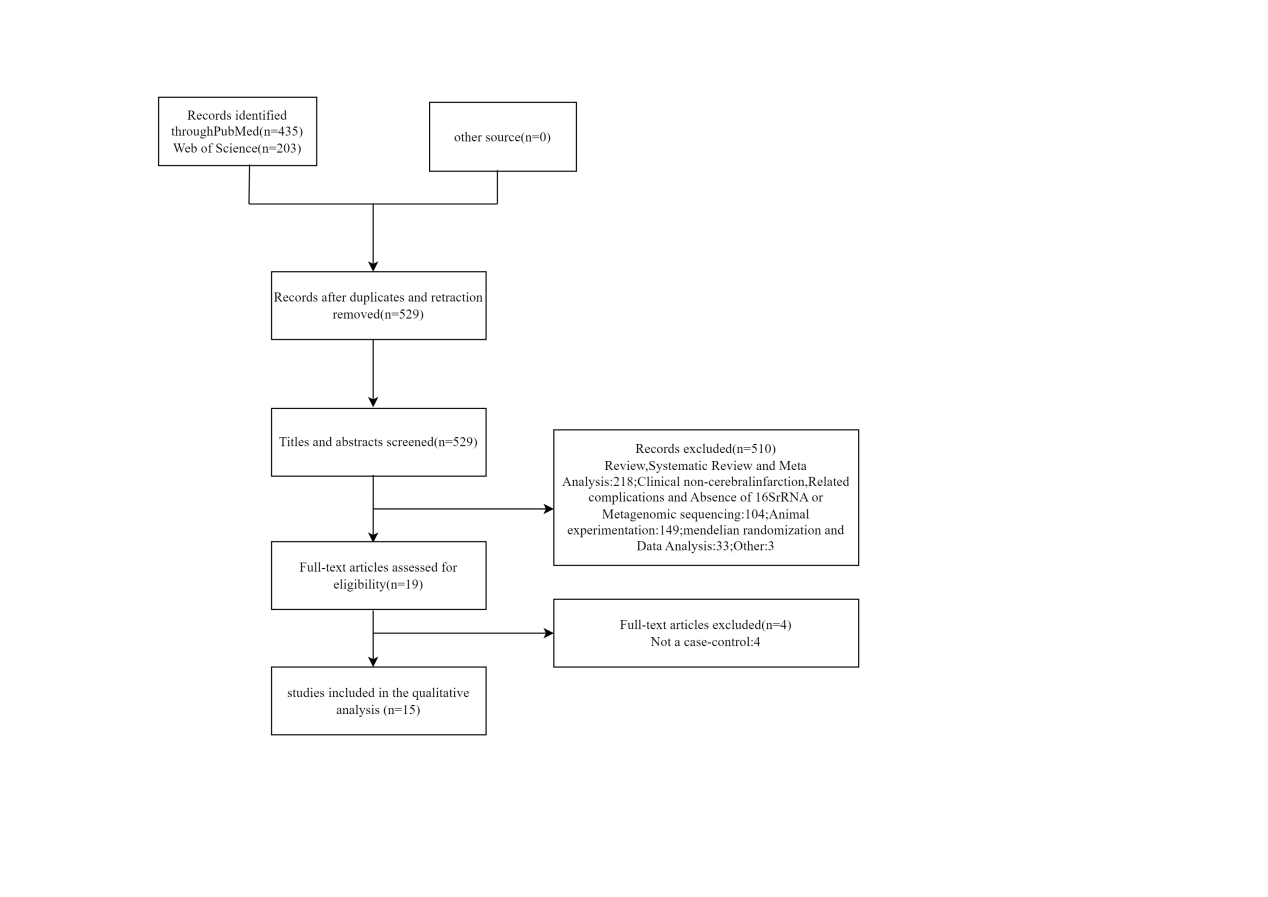


PubMed：((((((Ischemic Strokes*) OR (Ischaemic Stroke*)) OR (Acute Ischemic Stroke*)) OR (Cryptogenic Ischemic Stroke*)) OR (Cryptogenic Embolism Stroke*)) OR (Cryptogenic Stroke*)) AND (((((((((((Enteric Microbiota*) OR (Enteric Microflora Flora*)) OR (Gut Microflora)) OR (Gut Flora)) OR (Gut Microbiome*)) OR (Gut Microbiota*)) OR (Intestinal Microbiome*)) OR (Intestinal Microflora)) OR (Intestinal Flora)) OR (Intestinal Microbiota*)) OR (Enteric Bacteria))

Web of science：

#1 (((((TS=(Ischemic Strokes*)) OR TS=(Ischaemic Stroke*)) OR TS=(Acute Ischemic Stroke*)) OR TS=(Cryptogenic Ischemic Stroke*)) OR TS=(Cryptogenic Embolism Stroke*)) OR TS=(Cryptogenic Stroke*)

#2 ((((((((((TS=(Enteric Microbiota*)) OR TS=(Enteric Microflora Flora*)) OR TS=(Gut Microflora)) OR TS=(Gut Flora)) OR TS=(Gut Microbiome*)) OR TS=(Gut Microbiota*)) OR TS=(Intestinal Microbiome*)) OR TS=(Intestinal Microflora)) OR TS=(Intestinal Flora)) OR TS=(Intestinal Microbiota*)) OR TS=(Enteric Bacteria)

#3 #1 AND #2
